# Small molecule, big impact: H_2_S contributes to pathogen-induced stomatal closure

**DOI:** 10.1093/plphys/kiaf530

**Published:** 2025-10-21

**Authors:** Josephine H R Maidment, Anna Moseler

**Affiliations:** Assistant Features Editor, Plant Physiology, American Society of Plant Biologists; PHIM Plant Health Institute, Univ Montpellier, INRAE, CIRAD, Institut Agro, IRD, Montpellier 34980, France; Centre de Biologie Structurale, INSERM, CNRS, Université de Montpellier, Montpellier 34090, France; Assistant Features Editor, Plant Physiology, American Society of Plant Biologists; INRES-Chemical Signalling, University of Bonn, Bonn 53117, Germany

Plants are sessile organisms and must integrate information from a range of external stimuli to respond appropriately to their environment. Stomata form a key interface between the plant and its environment. They are dynamic pores, flanked by a pair of specialized guard cells found predominantly on the lower surface of plant leaves. Their aperture is carefully controlled to balance water loss with the uptake of carbon dioxide for photosynthesis (reviewed in Lawson and Leakey (2024)). Stomatal pores are also exploited by pathogenic microbes to enter a host plant (reviewed in Hou et al. (2024)). Closure of stomata to restrict pathogen entry is one of a suite of early defence responses activated by cell surface receptors following recognition of molecular signatures of pathogen attack. One such cell surface receptor is FLS2 (FLAGELLIN-SENSING 2), which detects an epitope of bacterial flagellin known as flg22 (Gómez-Gómez and Boller 2000). Activation of cell surface receptors such as FLS2 leads to production of apoplastic reactive oxygen species (ROS) via RBOHD (RESPIRATORY BURST OXIDATIVE HOMOLOGUE D), a subsequent cytosolic ROS burst, an influx of calcium ions into the cytoplasm (reviewed in Ryu et al. (2025)), and stomatal closure. The regulation of stomatal dynamics during pathogen infection is highly complex. Some pathogens have evolved mechanisms to counter plant defences and promote reopening of stomata, increasing potential for pathogen entry. In addition, when infection is already established, stomatal closure can promote the proliferation of the pathogen by increasing humidity in the mesophyll (Kemppinen et al. 2025; Hou et al. 2024). While much is known about the different pathways and components involved, it is evident that much remains to be discovered.

Hydrogen sulfide (H_2_S) is a gaseous signaling molecule involved in plant responses to environmental stresses (reviewed in Zhou et al. (2025)). While H_2_S is produced in different subcellular compartments, the breakdown of *L*-cysteine by DES1 (*L*-CYSTEINE DESULFHYDRASE 1) is a major source of cytosolic H_2_S (Álvarez et al. 2010). H_2_S contributes to oxidative stress tolerance by promoting the activity of antioxidant enzymes. Furthermore, H_2_S reacts with oxidized thiol groups of cysteine residues in proteins, leading to protein persulfidation. This post-translational modification can affect the structure, activity, or localization of a protein and protects proteins from irreversible oxidation (reviewed in Moseler et al. (2024)). Previous work has shown that H_2_S persulfidates key enzymes involved in guard cell signaling pathways, such as RBOHD (Shen et al. 2020). Furthermore, H_2_S regulates ion transport and accumulation of signaling molecules, such as nitric oxide, in guard cells, ultimately contributing to stomatal closure (Papanatsiou et al. 2015; Scuffi et al. 2014). However, compared with molecules such as ROS and Ca^2+^, less is known about the precise molecular mechanism of how cytosolic H_2_S contributes to plant defence.

In a recent study published in *Plant Physiology*, Scuffi et al. (2025) dissect the role of cytosolic H_2_S in pathogen-induced stomatal closure and integrate cytosolic H_2_S into the stomatal signaling network (Figure). In an initial experiment, the authors explored the function of DES1 as the main source of cytosolic H_2_S. Using the null mutants *des1-1* and *des1-2*, the authors analyzed how absence of DES1 impacts stomatal immunity. They showed that stomata of the 2 mutants do not respond to pathogens such as *Pseudomonas syringae* pv. *tomato* DC3000 nor to pathogen-derived molecules such as flg22 or the bacterial elongation factor Tu peptide 18 (elf18). Exogenous application of H_2_S, however, restored stomatal response to flg22 in the *des1* mutant, suggesting that H_2_S is indispensable for the stomatal immune response. Interestingly, while *des1* mutants show impaired stomatal closure, they were less susceptible to *Pst* than WT plants. In the later stages of infection, *Pst* promotes hydration of the apoplast to support bacterial proliferation. By reducing apoplastic water availability, open stomata can contribute to overall *Pst* resistance. The finding that *des1* is less susceptible to *Pst* is consistent with reports that stomatal dynamics at later stages of infection are more important than at early stages of infection in determining the overall resistance phenotype (Kemppinen et al. 2025).

Because the first immune response after flg22 perception is an apoplastic, RBOHD-dependent oxidative burst, the authors assessed whether H_2_S is required to induce apoplastic H_2_O_2_ production. They showed that flg22-treated leaves of the *des1* mutant displayed a lower apoplastic ROS burst than flg22-treated wild-type (WT) leaves, indicating that DES1 is required in RBOHD-mediated redox signaling. After the apoplastic ROS burst, flg22 perception triggers a rise in cytosolic H_2_O_2_. To analyze whether H_2_S is necessary to induce cytosolic oxidation, Scuffi and colleagues expressed the H_2_O_2_ biosensor, roGFP2-Orp1, in the cytosol. Upon flg22 treatment, oxidation of the sensor was observed in both WT and *des1* mutant guard cells, albeit with a lower magnitude of the response in the *des1* background. Additionally, application of an H_2_S scavenger to WT plants abolished cytosolic oxidation, indicating that H_2_S is required to induce cytosolic H_2_O_2_ flux in guard cells. To further dissect whether the H_2_S-dependent cytosolic oxidation is dependent on RBOHD, the authors expressed the H_2_O_2_ biosensor in the *rbohd* mutant. Incubation with the H_2_S donor GYY4137 induced an oxidation in the cytosol of both the WT and *rbohd* mutant, suggesting that H_2_S-induced cytosolic oxidation in guard cells does not require RBOHD.

Next, Scuffi and colleagues investigated the impact of H_2_S on Ca^2+^ fluxes, another part of the guard cell signaling network. The authors applied the H₂S donor to Arabidopsis epidermal peels, alongside calcium chelators BAPTA-AM and EGTA to selectively disrupt calcium pools in the cytosol and apoplast, respectively. Intriguingly, chelation of either calcium source prevented H₂S-induced stomatal closure, implicating Ca²⁺ fluxes as essential mediators of H₂S signaling in guard cell immunity.

In summary, Scuffi et al. (2025) add a new layer to the complex signaling network underlying stomatal defence by placing H_2_S and its cytosolic source DES1 as key players in stomatal immunity. The authors showed that H_2_S and DES1 are involved in the biotic stress response by modulating apoplastic and cytosolic H_2_O_2_ production and that Ca^2+^ availability is required for H_2_S-mediated stomatal closure. Further dissection of this cross-talk could illuminate how plants integrate diverse signals to finely tune immune outputs at the cellular interface with the environment. Open questions remain such as how DES1 is activated and what are the targets of H_2_S-mediated protein persulfidation and the impact of this posttranslational modification on enzyme activities. Additionally, the source of ROS that causes cytosolic oxidation is so far unknown. The insights of this study, however, pave the way to further study redox-mediated immune responses.

**Figure:**
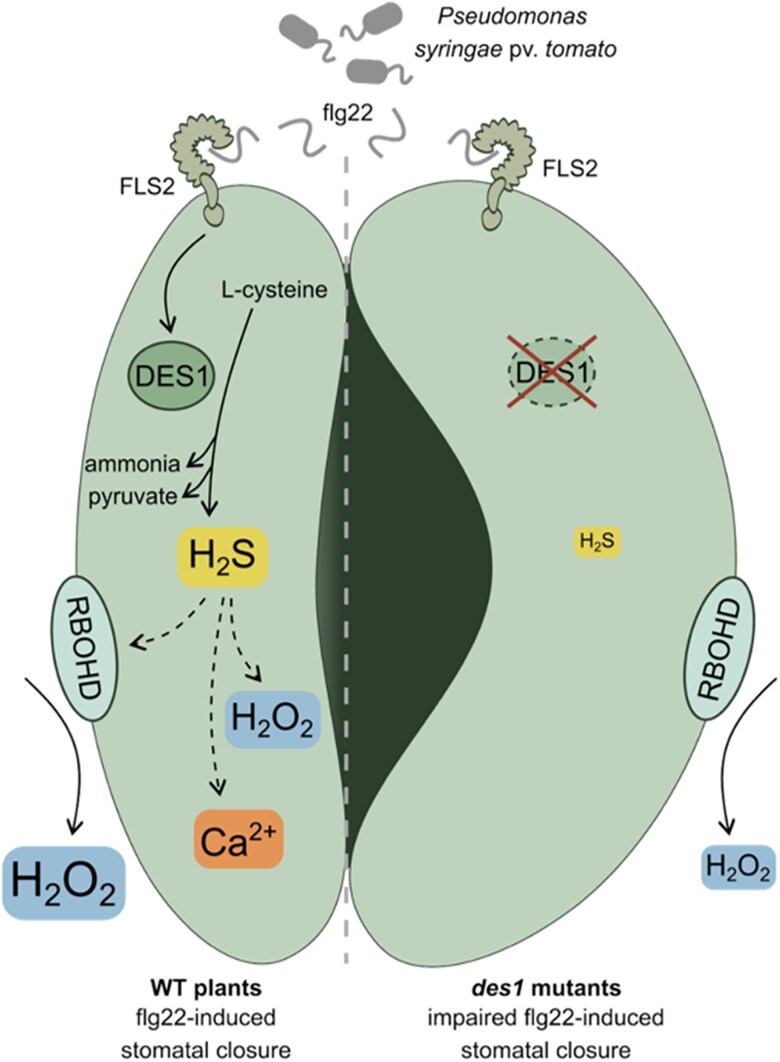
Working model of DES1 and H_2_S involvement in flg22-induced stomatal closure (modified from Scuffi et al. (2025)). Bacterial flg22 (e.g. from *Pseudomonas syringae* pv. *tomato*) is recognized by the cell surface receptor FLS2. Activation of FLS2 positively regulates DES1, which breaks down *L*-cysteine into ammonia, pyruvate, and H_2_S. DES1 is partially required to activate RBOHD for apoplastic ROS production. H_2_S induces accumulation of cytosolic H_2_O_2_. In *des1* mutants, production of cytosolic H_2_S and subsequent flg22-induced stomatal closure are compromised.

## Recent related articles in *Plant Physiology*:

- Kemppinen et al. (2025) explored how stomatal opening and closure affect plant resistance to *Pseudomonas syringae*.
- Huang et al. (2025) described how glycolate oxidase persulfidation regulates ROS production.
- Pantaleno et al. (2023) demonstrated that a mitochondrial H_2_S donor induces stomatal closure.

## Data Availability

No new data included in this article.

## References

[kiaf530-B1] Álvarez C, Calo L, Romero LC, García I, Gotor C. An O-acetylserine(thiol)lyase homolog with L-cysteine desulfhydrase activity regulates cysteine homeostasis in Arabidopsis. Plant Physiol. 2010:152(2):656–669. 10.1104/pp.109.147975 19955263 PMC2815857

[kiaf530-B2] Gómez-Gómez L, Boller T. FLS2: an LRR receptor-like kinase involved in the perception of the bacterial elicitor flagellin in Arabidopsis. Mol Cell. 2000:5(6):1003–1011. 10.1016/S1097-2765(00)80265-8 10911994

[kiaf530-B3] Hou S, Rodrigues O, Liu Z, Shan L, He P. Small holes, big impact: stomata in plant-pathogen-climate epic trifecta. Mol Plant. 2024:17(1):26–49. 10.1016/j.molp.2023.11.011 38041402 PMC10872522

[kiaf530-B4] Huang J, Wu K, Li X, Zeng X, Luo Y, Zhang Z, Peng X. Glycolate oxidase persulfidation mediates the salicylic acid-modulated GC switch to regulate photorespiratory H_2_O_2_ levels. Plant Physiol. 2025:198(4):kiaf369. 10.1093/plphys/kiaf369 40825035

[kiaf530-B5] Kemppinen J, Pollmeier M, Ehonen S, Brosché M, Sierla M. Water immunity overrides stomatal immunity in plant resistance to *Pseudomonas syringae*. Plant Physiol. 2025:198(1):kiaf127. 10.1093/plphys/kiaf127 PMC12063527 40173409

[kiaf530-B6] Lawson T, Leakey ADB. Stomata: custodians of leaf gaseous exchange. J Exp Bot. 2024:75(21):6677–6682. 10.1093/jxb/erae425 39545386 PMC11565196

[kiaf530-B7] Moseler A, Wagner S, Meyer AJ. Protein persulfidation in plants: mechanisms and functions beyond a simple stress response. Biol Chem. 2024:405(9–10):547–566. 10.1515/hsz-2024-0038 39303198

[kiaf530-B8] Pantaleno R, Scuffi D, Costa A, Welchen E, Torregrossa R, Whiteman M, García-mata C. Mitochondrial H_2_S donor AP39 induces stomatal closure by modulating guard cell mitochondrial activity. Plant Physiol. 2023:191(3):2001–2011. 10.1093/plphys/kiac591 36560868 PMC10022628

[kiaf530-B9] Papanatsiou M, Scuffi D, Blatt MR, García-mata C. Hydrogen sulfide regulates inward-rectifying K^+^ channels in conjunction with stomatal closure. Plant Physiol. 2015:168(1):29–35. 10.1104/pp.114.256057 25770153 PMC4424018

[kiaf530-B10] Ryu H, Choi S, Cheng M, Koo B-K, Kim EY, Lee H-S, Lee D-H. Flagellin sensing, signaling, and immune responses in plants. Plant Commun. 2025:6(7):101383. 10.1016/j.xplc.2025.101383 40400167 PMC12281300

[kiaf530-B11] Scuffi D, Álvarez C, Laspina N, Gotor C, Lamattina L, García-mata C. Hydrogen sulfide generated by L-cysteine desulfhydrase acts upstream of nitric oxide to modulate abscisic acid-dependent stomatal closure. Plant Physiol. 2014:166(4):2065–2076. 10.1104/pp.114.245373 PMC4256879 25266633

[kiaf530-B12] Scuffi D, Pantaleno R, Schiel P, Niemeier J-O, Costa A, Schwarzländer M, Laxalt AM, García-mata C. Hydrogen sulfide modulates flagellin-induced stomatal immunity. Plant Physiol. 2025:199:2025–202. 10.1101/2025.02.14.638267 41432548

[kiaf530-B13] Shen J, Zhang J, Zhou M, Zhou H, Cui B, Gotor C, Romero LC, Fu L, Yang J, Foyer CH, et al Persulfidation-based modification of cysteine desulfhydrase and the NADPH oxidase RBOHD controls guard cell abscisic acid signaling. Plant Cell. 2020:32(4):1000–1017. 10.1105/tpc.19.00826 32024687 PMC7145499

[kiaf530-B14] Zhou M, Xie Y, Vanbreusegem F, Huang J. Hydrogen sulfide and protein persulfidation in plant stress signaling. J Exp Bot. 2025:76(13):3738–3757. 10.1093/jxb/eraf100 40059712

